# Calcium Alkali Syndrome Treated With Hemodialysis

**DOI:** 10.7759/cureus.13749

**Published:** 2021-03-07

**Authors:** Barbara M Parker

**Affiliations:** 1 Clinical Pharmacy, AdventHealth Orlando, Orlando, USA; 2 Clinical Pharmacy, Rockledge Regional Medical Center, Rockledge, USA

**Keywords:** hypercalcemia, acute kidney injury

## Abstract

Malignancy, primary hyperparathyroidism, and vitamin D intoxication are the most common causes of hypercalcemia. Symptoms of hypercalcemia are nonspecific and require a plasma calcium level to diagnose. Undiagnosed hypercalcemia can cause renal failure long-term. Here, we describe a unique case of hypercalcemia resulting in acute kidney injury (AKI) secondary to overconsumption of calcium carbonate (Tums).

## Introduction

Hypercalcemia occurs when the influx of calcium to the intracellular space from bone resorption and intestinal calcium absorption exceeds the rate at which it can be excreted by the kidney [1]. Calcium alkali syndrome, resulting in hypercalcemia, occurs when the intestine absorbs an excessive amount calcium from calcium-containing antacids or phosphate binders [1]. Calcium supplementation doses of above 4 g are generally associated with calcium alkali syndrome, but daily doses of 1-1.5 g (normal dietary recommendations) have also been reported [2]. We present a unique case of a patient with severe, symptomatic hypercalcemia and acute renal failure not due to malignant disease, primary hyperparathyroidism, or vitamin D intoxication, but rather from calcium alkali syndrome (overconsumption of antacids).

## Case presentation

A 66-year-old morbidly obese woman presented to the ED with generalized weakness over the last seven days, oliguria, poor appetite, and thirst. Her past medical history included hypertension, hyperlipidemia, congestive heart failure, pulmonary hypertension, restrictive lung disease, morbid obesity, and type 2 diabetes mellitus. She had no history of cancer or multiple myeloma, or renal disease as evident from past admissions.

**Figure 1:**
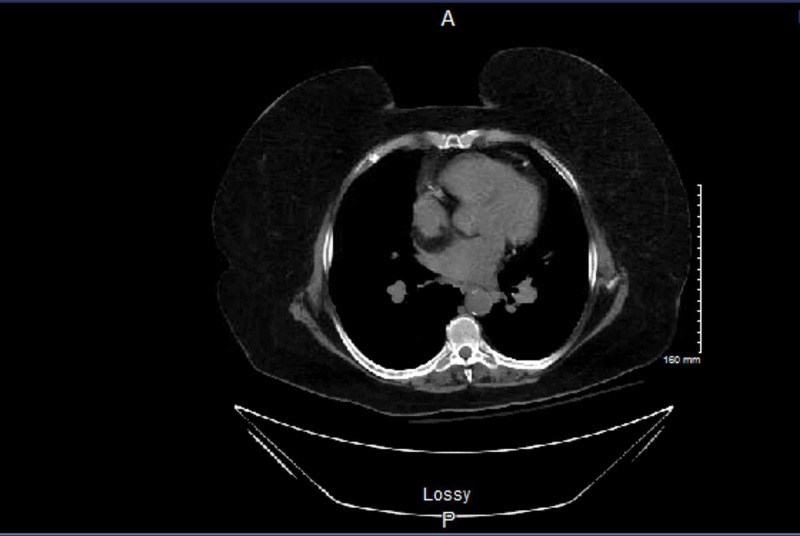
CT abdomen showing an 18 mm cyst upper pole right kidney with a parapelvic cyst measuring 18 mm x 18 mm.

**Figure 2:**
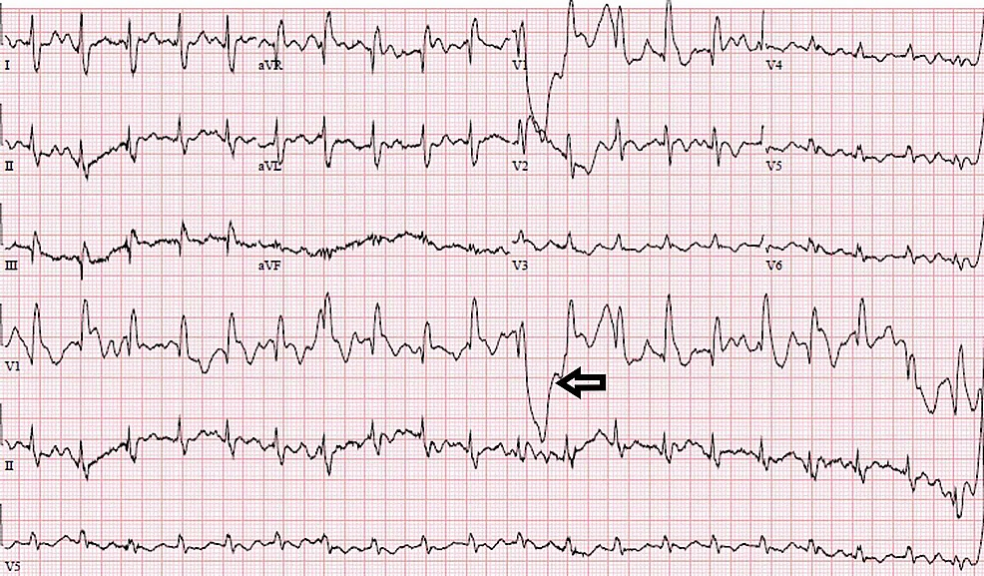
EKG showing intraventricular block. EKG, electrocardiogram

Upon presentation to the ED, she was found to have a serum creatinine of 5 mg/dL (her baseline was 0.5 mg/dL) and a serum calcium level of 23.1 mg/dL (normal reference range: 8-10.4 mg/dL) and ionized calcium of 3.23 mg/dL (normal reference range: 4.23-5.11 mg/dL). Her albumin was 3.9 g/dL, within normal range, indicating true hypercalcemia. CT of her abdomen and pelvis revealed an 18 mm cyst in upper pole of the right kidney with a parapelvic cyst measuring 18 mm x 18 mm (Figure 1). Chest X-ray (CXR), CT of head also did not show any significant pathology. Her other labs included a white blood cell (WBC) 14.14, hemoglobin 11.8 g/dL, platelet 427,000 platelets per microliter, sodium 131 mEq/L, potassium 3.8 mEq/L, chloride 89 mEq/L, carbon dioxide 36 mEq/L (normal reference 20-32 mEq/L), blood urea nitrogen (BUN) 62 mg/dL, magnesium 3 mEq/L, aspartate aminotransferase (AST) 71 IU/L, alanine aminotransferase (ALT) 46 IU/L, troponin 39 ng/mL, B-type natriuretic peptide (BNP) 435 pg/mL, international normalized ratio (INR) less than 0, and partial thromboplastin time (PTT) more than 212 s. Her electrocardiogram (EKG) showed intraventricular right bundle branch block (Figure 2). Her vitals included a temperature of 97.8 degrees Fahrenheit, pulse of 72 beats/min, and blood pressure of 160 over 83 millimeters of mercury. A hypercalcemia work-up was done which included parathyroid hormone (PTH), PTH-related protein, and serum protein electrophoresis. PTH was found to be low at 13.9 pg/mL (normal range 15-75 pg/mL) and PTH-related protein (PTHrP) was less than 2 pmol/L, normal. Serum immunofixation showed no monoclonal band, and serum and urine protein electrophoresis were unremarkable. Viral hepatitis serologies were negative. Thyroid stimulating hormone (TSH) was 3.14 uIU/mL, normal. Vitamin D 25-hydroxy was normal, 50.3 ng/mL.

**Figure 3:**
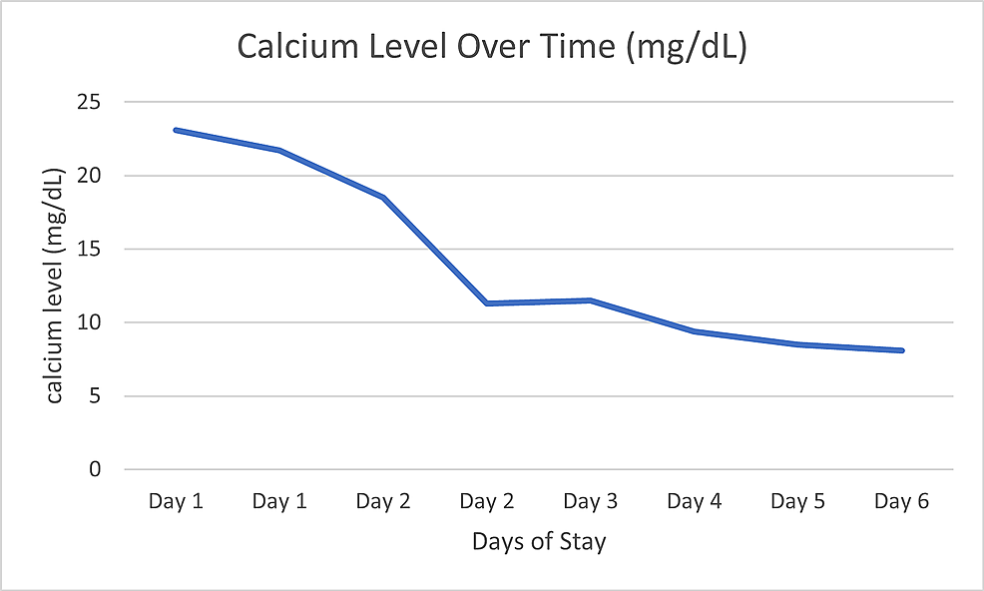
Changes over time of calcium level after administration of Zometa and Miacalcin and dialysis sessions on days two and three.

**Figure 4:**
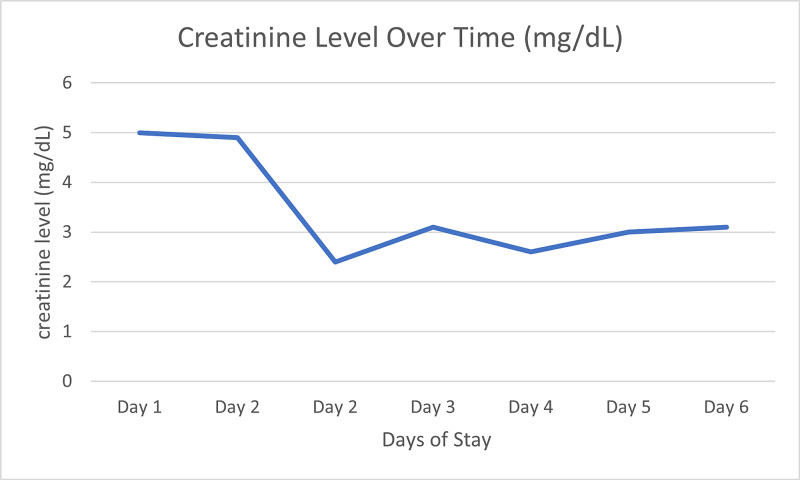
Changes over time of serum creatinine after administration of Zometa and dialysis sessions on days two and three.

Treatment was initiated with fluid resuscitation (1 L normal saline bolus), the bisphosphonate Zometa (Sagent Pharmaceuticals, Schaumburg, IL), and Miacalcin (Mylan, Rockford, IL). A temporary dialysis catheter was placed and dialysis treatment was initiated 12 h later (day two), and again on day three due to a persistently elevated serum calcium level of 11.5 mg/dL. Dialysis was necessary as Zometa and Miacalcin were not working fast enough to ensure a positive outcome alone. In addition, there was concern for acute tubular necrosis due to Zometa in our patient with a creatinine clearance of less than 30 mL/min (Figures 3-4, Table 1). The patient’s serum creatinine and calcium levels improved by day six and patient was discharged home with outpatient follow-up.

**Table 1:** Calcium and creatinine level changes over time from day one to day six of admission.

	Calcium level (mg/dL)	Creatinine (mg/dL)
Day 1	23.1	5
Day 1	21.7	5
Day 2	18.5	4.9
Day 2	11.3	2.4
Day 3	11.5	3.1
Day 4	9.4	2.6
Day 5	8.5	3
Day 6	8.1	3.1

## Discussion

Calcium alkali syndrome occurs when too much calcium is absorbed from the intestine due to excess intake of calcium-containing antacids or phosphate binders [1]. It has been speculated that alkalosis and hypercalciuria in combination lead to hypercalcemia with renal parenchymal calcification and tubular damage [1]. Hypercalcemia does not occur when excessive amounts of calcium is taken in the absence of alkali, or when alkali is taken without calcium [1]. Most patients present with asymptomatic hypercalcemia, but in our case, the patient presented with symptoms (weakness, polyuria, polydipsia). When PTH is high in the presence of hypercalcemia, then the cause of the hypercalcemia is usually primary or tertiary hyperparathyroidism [3]. If the PTH level is suppressed (as was mildly in our case), one most often suspects malignancy [3]. CTs and CXR were insignificant in our patient for any malignancy. After interviewing the patient, she stated she was taking some calcium supplements and consumed an entire bottle of Tums (calcium carbonate) daily, which was the most probable explanation after hyperparathyroidism and malignancy were ruled out.

Subcutaneous (4 units per kilogram) Miacalcin, a short acting therapy, is accompanied by a longer acting therapy (Aredia or Zometa) when a patient presents with hypercalcemia symptoms [4-5]. Miacalcin’s acute effect on decreasing high plasma levels of calcium ceases after 24-48 h due to the development of drug tolerance over time by the kidneys [5]. Unfortunately, our choices for long-acting agents were not good as patient presented with acute kidney injury (AKI) and bisphosphonates (Aredia and Zometa) can exacerbate the severity of AKI in the setting of hypercalcemia [5-7]. There is evidence that both drugs renally accumulate and are retained in the renal cortex and medulla, likely via endocytosis [5, 7]. Aredia was not available at the time, but Zometa was administered considering benefit of each of these medications. A head-to-head comparison of Aredia and Zometa in two randomized controlled trials demonstrated that Zometa is superior to Aredia in both efficacy and duration of response [8]. Although not recommended for creatinine clearance of less than 30 mL/min, an alternative strategy is to administer Zometa 4 mg dosage over 23 hours instead of 30 minutes [7-8]. It can also be given at half its dose over 15 minutes [7-8]. Xgeva (a monoclonal antibody) or Clasteon (a bisphosphonate) could have also been considered as agents for treatment of hypercalcemia with significant renal impairment [7-10]. As Zometa takes two to three days for maximum efficacy, it is not the fastest means to correct life-threatening hypercalcemia, as opposed to hemodialysis [5, 7, 11]. Dialysis was required in this patient, as despite administration of both Miacalcin and Zometa, the patient’s calcium remained at a life-threatening level. Severe hypercalcemia causes muscle flaccidity, AKI, brain dysfunction with obtundation or coma, arrhythmia, and cardiac arrest [7, 10-11]. Hemodialysis is rarely used in cases of hypercalcemia due to its invasive nature but was required to save our patient’s life [4, 7, 11].

Ninety percent of all hypercalcemia cases are due to hyperparathyroidism and malignancy, leaving 10% of cases due to other causes [1, 3, 8-9, 12-14]. Hypercalcemia due to calcium-alkali syndrome is very uncommon but needs to remain a diagnostic consideration when other etiologies are ruled out. A thorough history including medication and supplement review is required.

## Conclusions

Instances of hypercalcemia due to causes other than hyperparathyroidism and malignancy are rare. A comprehensive workup to rule out any of the following contributing conditions: parathyroid hormone mediated hypercalcemia, malignancy, vitamin D intoxication, endocrine disorders, and drug-induced hypercalcemia is needed. Bisphosphonate dosing adjustments should be made in hypercalcemic patients with severe renal impairment. Hemodialysis is required with severe hypercalcemia refractory to treatments including aggressive volume resuscitation, Miacalcin, and bisphosphonate.

## References

[1] Ralston S, Coleman R, Fraser W (2004). Medical management of hypercalcemia. Calcif Tissue Int.

[2] Patel AM, Adeseun GA, Goldfarb S (2013). Calcium-alkali syndrome in the modern era. Nutrients.

[3] Turner J (2017). Hypercalcaemia - presentation and management. Clin Med (Lond).

[4] Deftos LJ (1996). Hypercalcemia: mechanisms, differential diagnosis, and remedies. Postgrad Med.

[5] Adam Z, Vorlícek J, Tomíska M (1994). Základy farmakologie bisfosfonátů a jejich pouzití pri hyperkalcémii [The pharmacology of biphosphonates and their use in hypercalcemia]. Vnitr Lek.

[6] Ellis S, Tsiopanis G, Lad T (2018). Risks of the 'Sunshine pill' - a case of hypervitaminosis D. Clin Med (Lond).

[7] Hirschberg R (2012). Renal complications from bisphosphonate treatment. Curr Opin Support Palliat Care.

[8] Minisola S, Pepe J, Piemonte S, Cipriani C (2015). The diagnosis and management of hypercalcaemia. BMJ.

[9] Dahmani O, Sophoclis C, Kebir M, Bouguern D, Sakho A, Demarchi P (2017). Denosumab for the treatment of bisphosphonate resistant hypercalcemia in a hemodialysis patient. Saudi J Kidney Dis Transpl.

[10] Koike K, Fukami K, Morishige S (2009). [Case of acute kidney injury related to intravenous zoledronic acid in a patient with multiple myeloma]. Nihon Jinzo Gakkai Shi.

[11] Basok AB, Rogachev B, Haviv YS, Vorobiov M (2018). Treatment of extreme hypercalcaemia: the role of haemodialysis. BMJ Case Rep.

[12] Bilezikian JP (1988). Hypercalcemia. Dis Mon.

[13] Vergès B (2001). Hypercalcémie du sujet âgé [Hypercalcemia in the elderly]. Presse Med.

[14] Im H, Choi HM, Oh DJ, Kwon YE (2018). Severe acute kidney injury after single-dose injection of zoledronic acid in an elderly patient. Am J Ther.

